# Male recognition bias in sex assignment based on visual stimuli

**DOI:** 10.1038/s41598-022-12411-1

**Published:** 2022-05-17

**Authors:** Stefano Federici, Alessandro Lepri, Silvia Bacci, Francesco Bartolucci

**Affiliations:** 1 Department of Philosophy, Social and Human Sciences and Education, University of Perugia, Piazza G. Ermini 1, 06123 Perugia, Italy; 2 Myèsis, Research and Development Company, Rome, Italy; 3 Department of Statistics, Computer Science and Applications “G. Parenti”, University of Florence, Florence, Italy; 4 Department of Economics, University of Perugia, Perugia, Italy

**Keywords:** Psychology, Human behaviour, Evolution, Evolutionary theory

## Abstract

According to previous ethnomethodological and cognitive studies on sex assignment, if a figure has male sexual characteristics people are more likely to think it is a man than they think it is a woman when the figure has female sexual characteristics. This bias in favor of male attribution is strongly reinforced when a penis is apparent in human nude pictures. In our contribution, we reported findings of three experiments aimed at replicating previous studies by administering the Sex/Gender Attribution Test for Adult (SGAT-A) created by digitally morphing the bodies of one human male and one human female model into realistic pictures. We observed the sex attribution and response time of 1706 young adult participants. A cross-cultural comparison was also carried out with a sample of young adult Chinese students. Findings substantially reconfirmed those obtained in previous studies. The male external genitalia overshadow any other features that might rather suggest a female identity. Indeed, when male external genitalia were exposed, the odds of male sex attribution were 5.688 compared to 1.823 female attribution when female external genitalia were shown. Moreover, the shortest response times were observed with masculine stimuli. Evolutionary and cultural determinants of the male sex bias are also discussed.

## Introduction

According to the evolutionary psychological view of the human mind, adaptations are believed to occur at the level of psychological mechanisms rather than at the level of overt behavior^1^. This means that the common evolved architecture of the human mind does not contradict the different manifestations of behavior or psychology observed across individuals and cultures. In other words, different environmental inputs can result in different manifested outputs, while triggering the same underlying evolved psychology^1^. Therefore, we can assume that a universal evolved cognitive mechanism (reasoning, emotion, motivation, and motor control, whether the process that gives rise to it is conscious or unconscious, simple or complex, conscious or unconscious) underlies sex recognition and attribution, going beyond the individual and cultural differences in gender identity, gender expression, gender orientation/attraction, gender binary or gender spectrum/fluid, gender expression/presentation, cis- or trans-gender. In what follows, to avoid misunderstandings we use the expression “sex” to refer to a set of biological attributes that are associated with physical and physiological features usually categorized as female or male, although there is variation in the biological attributes, whereas we use the expression “gender” to refer to a range of characteristics used to distinguish between men and women and the masculine and feminine attributes culturally assigned to them^2–4^. It seems clear that all of the manifestations of gender have a common element: an individual’s ability to discriminate between biological sexes and their expressions. We have been hardwired to recognize a person’s sex from the clues that their gender expression and sexual characteristics show us of their biological identity, if the variation in individual and cultural differences in which gender manifests itself has not prevented human mate selection and sexual reproduction^5^.

Ito and Urland^6^ have highlighted that our brains form dichotomic categorization of gender with a staggering speed. Findings of electrocortical measures of attention to the gender of multiply categorizable individuals showed that sensitivity to gender information emerged slightly later than the sensitivity to race information seen as early as 122 ms. In addition, the amplitude of event-related brain potential components—which increases as a function of the extent to which attention is directed at some features of an external stimulus—was significantly larger when participants’ attention was directed to male than female stimuli.

The strength of categorizing individuals is shown not only by the speed and minimal sensory stimuli required for the brain to process group differences and the unconscious automaticity of such processes^6–8^, but by its presence also in very young children. By the age of three to four, children already group people by race and gender^9,10^. They focus on perceptually salient attributes in people (race, sex, age, and attractiveness) and, due to their poor cognitive ability, children categorize them in bipolar terms, incapable of processing all the internal qualities of individual differences^11^. These cognitive biases, although originating from cognitive human processes, nevertheless tend to be permeable to cultural environments that foster explicit structured schemes to make certain classifications perceptually salient^11,12^.

In a previous study, Federici and colleagues^13^, investigating what influence human sexual characteristics and gender-linked characteristics binaries have on cognitive processes of sex attribution in adults, found that the cultural stereotypes and prejudices that affect sex attribution might not just be a mere cultural product, but rather the consequence of evolved cognitive mechanisms “specialized for solving evolutionarily long-enduring adaptive problems and that these mechanisms have content-specialized representational formats, procedures, cues, and so on”^1^. Cognition and its products, the sex/gender representations, are the outcome of determinants both internal to the human organism (brain) and external (culture). The study makes evident that the representation of sex is given by the outcome of sexual characteristics that will fluctuate from those more typically natural/biological (sex) to those more typically cultural (gender), such as clothing or hair length. Moreover, Federici and colleagues, on the basis of their findings, also infer that the universal^14^ patriarchal and phallocentric cultural construction of sex/gender can be explained by an innate intuitive ability to easily grasp and learn these cultural meanings of gender identity. Freud’s version of the one-sex/gender model (having a penis means being a boy and not having a penis means being a girl) “is not only a patriarchal phallocentric elevation of a ‘biological fact’ into a cultural desideratum. It captures processes of psychological functioning, which reveal to us cognitive mechanisms at the origin of cultural biases”^13^. For instance, among other significant and remarkable results, the adult participants attributed male sex 86% of the time when the penis was shown, but only attributed female sex 67% of the time when the vulva was shown.

The Federici and colleagues’ study^13^ with adult participants was conducted administering the Sex/Gender Attribution Test for Adult (SGAT-A), designed by the authors (SF and AL). It includes 120 stimuli (pictures) of frontal human nudes created by combining parts of two original photographs of one male and one female model (Supplementary Table S16). For each stimulus randomly presented through an Internet platform, the participants were asked to assign the male or the female sex. In the present study we are interested in the replicability and comparison of responses to SGAT-A obtained by the first study^13^ with three other studies. In this way, we mean to verify the overall consistency of SGAT-A under similar experimental conditions. Through this, we aim to provide the scientific community with a research tool with which to investigate the cognitive processes underlying the person’s sex/gender recognition, ideally continuing the goals of Kessler and McKenna’s study^15^ and overcoming the limitations imposed by their unrealistic stimuli^13^.

In Experiment 1, the experimental design of Federici and colleagues was replicated by expanding the sample of participants. Participants were all Italian people.

In Experiment 2, Chinese participants were recruited in order to test the possible cultural effect on gender representation and, in particular, on the fact that the penis more than the vulva and the male sexual characteristics more than the female ones are significantly more salient in the sex attribution process^13,15^.

In Experiment 3, we have also examined the time taken by participants to attribute sex after each stimulus was displayed. Indeed, as Federici and colleagues^13^ have assumed, the cultural stereotypes and prejudices that affect gender attribution might be the consequence of cognitive biases, evolved to solve adaptive problems related to survival—namely, to avoid what is the greatest danger: an (angry) adult male. We expect this psychological mechanism to foster reliance on the availability heuristic (the ease with which instances come to mind), less cognitive effort, and fast thinking in male than female recognition^16^.

## Results

In this section we present the main results of our study. First, we investigate the presence of differences in sex attribution between sub-groups of individuals. Second, we describe how the presence (or the absence) of sexual characteristics may affect the sex attribution. Then, a special focus is devoted to the confidence in the sex attribution, the pleasantness of the co-presence of female and male characteristics, and the times taken to attribute sex according to the sexual characteristics displayed.

Results are detailed for each of the three experiments.

### Sex attribution and respondents’ individual characteristics

To investigate whether the sex of respondents (i.e., sex assigned at birth as male/female) affects the perception of the sex of a visual stimulus, a *z*-test was run to compare the proportion of “female” responses to the SGAT-A stimuli by female respondents with the proportion of “female” responses to the SGAT-A stimuli by male respondents (null hypothesis is about the equality of the two proportions and the alternative hypothesis is bidirectional). None of the three experiments provides sufficient evidence to reject the null hypothesis (Experiment 1: z = − 1.55, df = 885, p = 0.1213; Experiment 2: *z* = 0.75, df = 26, *p* = 0.4597; Experiment 3: z = 0.0267, df = 758, p = 0.9787; assumed equal variances). Thus, we can conclude in favor of no difference in the sex attribution between female and male respondents.

One-way between-subject ANOVAs were conducted to compare the effect of the other individual socio-demographic characteristics extracted from the questionnaire (such as, gender identity, level of education, political orientation, and religion orientation) and the sexual orientation measured through the Kinsey Scale^17^. In particular, these ANOVAs were based on the sex attribution, the confidence scale, and the pleasantness scale for Experiment 1, on sex attribution and the confidence scale for Experiment 2, and on the sex attribution and response times for Experiment 3. ANOVA tests did not reveal any significant effect with regard to sex attribution (Supplementary Tables S3, S12, and S13), with the only exception for Experiment 1 of a weak but significant effect due to the political orientation (*p* = 0.037; 41% of female attribution by left-wing and center people vs 39% of female attributions by right-wing people).

In Experiment 1, differences were found between participants without political opinions (2.68 vs 3.22 of left-wing respondents), and those with lower educational level (2.74 for respondents with high school diploma vs 3.11 for bachelor and 3.19 for master degree graduates) in the confidence scale as well as in the pleasantness in sex attribution (Supplementary Tables S4 and S5, respectively). Statistically but not substantial significant differences were also found in the confidence scale as well as in the pleasantness in sex attribution with regard to gender identity and sexual orientation.

We also evaluated the measure’s reliability regarding internal consistency calculated with Cronbach’s alpha. For the sex attribution (male/female) based on the 120 stimuli, the coefficient was good in Experiments 1 and 3 (α = 0.90; 95% CI = [0.860; 0.927] and α = 0.86; 95% CI = [0.839; 0.875], respectively), while acceptable in Experiment 2 (α = 0.70; 95% CI: [0.475; 0.805]). The coefficient was excellent (α = 0.99) both for the confidence scale (Experiment 1: 95% CI = [0.991; 0.993]; Experiment 2: 95% CI: [0.964; 0.993]) and the pleasantness scale (Experiment 1: 95% CI = [0.995; 0.996]).

### The effect of sexual characteristics on sex attribution

The effect of sexual characteristics of the displayed pictures on sex attribution was observed with descriptive analyzes in the three experiments and estimated in experiments 1 and 3 with mixed logit models for the following binary response variables (mixed logit models have not been estimated in Experiment 2 due to the small sample size): (i) *Y*_1*ij*_ = 1 if individual *i* attributed male sex to stimulus *j*, and 0 if he/she attributed female sex (*i* = 1, …, *I*, with *I* = 887 in Experiment 1 and *I* = 779 in Experiment 3; *j* = 1, …, 120); (ii) *Y*_2*ij*_ = 1 if sex attributed by individual *i* to stimulus *j* was coherent with the type of stimulus (i.e., masculine vs. feminine), and 0 if the attribution was incoherent (*i* = 1, …, *I*, with *I* = 887 in Experiment 1 and *I* = 779 in Experiment 3; *j* = 1, …, 120). Both response variables were regressed on the binary sexual variables characterizing the pictures, that is, hair (1 if long), face (1 if feminine), hips (1 if wide), body hair (1 if absent), breast (1 if present), vulva (1 if present). Note that all stimuli are active (i.e., equal to 1) when referring to female characteristics. For each response variable, we estimated four mixed logit models, according to whether the model in the picture wore unisex jeans (hiding hips and external genitalia) and/or unisex t-shirt (hiding breasts/chest). Thus, we defined the following models:

- Stimuli without jeans and t-shirt variables (model of type 00): all the six sexual characteristics are visible.
- Stimuli without jeans and with t-shirt variables (model of type 01): breast/chest are not visible.
- Stimuli with jeans and without t-shirt (model of type 10): vulva/penis and hips are not visible.
- Stimuli with jeans and with t-shirt (model of type 11): breast/chest, vulva/penis, and hips are not visible.

Models were estimated through the maximum likelihood approach with Laplace approximation, using function glmer of R package lme4^18,19^.

Details on the descriptive analyses and on the estimated models are provided in the following for each experiment.

#### Experiment 1

Respondents attributed male sex to 60% of the stimuli. More precisely, the percentage of participants attributing male sex was 84.4% when the stimulus had more masculine variables (N = 47 stimuli with male variables > 3), 35.3% when the stimulus had more feminine variables (N = 47 stimuli with female variables > 3), and 60.1% when the stimulus was neutral (N = 26 stimuli with balanced co-presence of male and female variables); χ^2^ (2, *N* = 106,212) = 20,911, p < 0.001. In the case of 20 neutral stimuli, consisting of 10 neutral/female pictures, where the vulva is uncovered or the female face is shown when the external genitalia are covered by jeans, and 10 neutral/male pictures, where the penis is uncovered or the female face is shown when the external genitalia are covered, 83.3% of the neutral/male stimuli compared to 63.2% of the neutral/female stimuli were congruently attributed; χ^2^ (1, *N* = 106,440) = 5507, p < 0.001. When the penis was exposed in a picture, the participants attributed male sex significantly more often (87.7%) than female sex when the vulva was exposed (69.6%); χ^2^ (1, *N* = 106,440) = 28,893, p < 0.001).

All the sexual characteristics significantly contribute to the attribution of sex (Supplementary Table S6). As expected, the presence of female characteristics reduces the probability of assigning male sex (signs of regression coefficients are negative). When all sexual characteristics are visible (Model 00), the strongest effect is due to the presence of vulva (OR = 0.030), followed by a feminine face (OR = 0.250) and the presence of breasts (OR = 0.281); the presence of long hair provides the smallest contribution (OR = 0.849). When breasts are covered by t-shirt (Model 01), results are like those of Model 00. When the external genitalia are covered by jeans (Model 10 and Model 11), the face assumes a relevant role (OR = 0.092 in Model 10 and OR = 0.005 in Model 11), together with the presence of breasts (Model 10) and the absence of body hair (Model 11). At the same time, the effect of long hair (gender-linked of secondary sexual characteristics) increases when the number of primary sexual characteristics (penis/vulva) reduces (OR = 0.740 in Model 10 and OR = 0.525 in Model 11) (Supplementary Tables S6 and S7).

#### Experiment 2

Chinese respondents attributed male sex to 58.1% of the stimuli. More precisely, the percentage of participants attributing male sex was 82.0% when the stimulus had more masculine variables, 34.3% when the stimulus had more feminine variables, and 57.8% when the stimulus was neutral; χ^2^ (2, *N* = 3598) = 658.68, p < 0.001. When the penis was exposed in a stimulus, the participants attributed male sex significantly more often (88.4%) than female sex when the vulva was exposed (73.3%), χ^2^ (1, *N* = 2878) = 1114, p < 0.001). Considering neutral stimuli as neutral/female and as neutral/male, 81.4% of neutral/male pictures against 65.3% of the neutral/female pictures were congruently attributed χ^2^ (1, *N* = 3600) = 119.54, p < 0.001.

As outlined above, mixed logit models have not been estimated in Experiment 2 due to the small sample size.

#### Experiment 3

Respondents attributed male sex to 58.8% of the pictures. More precisely, the percentage of participants attributing male sex was 82.7% when the stimulus had more masculine variables, 35.1% when the stimulus had more feminine variables, and 58.6% when the stimulus was neutral; *χ*^2^ (2, *N* = 93,368) = 17,077, p < 0.001. When the penis was exposed in a picture, the participants attributed male sex significantly more often (84.3%) than they attributed female sex when the vulva was exposed (68.5%), χ^2^ (1, *N* = 74,683) = 21,344, p < 0.001). Considering the 20 neutral stimuli as neutral/female and as neutral/male, 80.1% of masculine pictures as opposed to 62.6% of feminine pictures were correctly attributed, *χ*^2^ (1, *N* = 94,560) = 3526.3, p < 0.001.

Estimates of fixed effects of the mixed logit models (Supplementary Tables S14 and S15) returned values in line with those obtained in Experiment 1 (“The effect of sexual characteristics on sex attribution”), except for the Model 01 (breast/chest are not visible) where the presence of vulva is here significant (p = < 0.05), although weak (OR = 0.944) with a negative effect (Supplementary Table S15).

#### Confidence in sex attribution

Confidence in sex attribution was investigated in experiments 1 and 2. If we consider the conditions where the penis was exposed, 27.1% of the Italians (Experiment 1) and 27.1% of Chinese participants gave a certainty score of 7, indicating they had no doubt about the sex attributed to the stimulus. By contrast, when the vulva was exposed, 20% of the Italian (Experiment 1) and 19.3% of the Chinese participants gave a certainty score of 7 (*χ*^2^ [1, *N* = 85,152] = 596.45, p < 0.001; *χ*^2^ [1, *N* = 2880] = 24.015, p < 0.001, respectively). When participants attributed female sex, 78.6% of Italians (Experiment 1) and 80.6% of Chinese declared they were uncertain (scores 1–6), but when participants attributed male sex, 73.2% of Italians (Experiment 1) and 74.5% of Chinese indicated uncertainty; *χ*^2^ (1, *N* = 106,212) = 408.19, p < 0.001 and *χ*^2^ (1, *N* = 3598) = 16.067, p < 0.001, respectively.

#### Pleasantness in the co-presence of male and female sexual characteristics

Of the participants in Experiment 1, 38.6% found the pictures with the 26 neutral stimuli (balanced co-presence of male and female variables and no clothing) totally unpleasant (score = 1). By contrast, 31.3% and 30.5% of the participants found the stimulus totally unpleasant when it had unbalanced sexual variables with a prevalence of, respectively, female or male characteristics, *χ*^2^ (2, *N* = 106,440) = 492.55, p < 0.001. In addition, 3.4% of the participants found the neutral stimuli as pleasant (score = 7) with respect to 6.2% of female stimuli and 7.8% of male stimuli, *χ*^2^ (2, *N* = 106,440) = 494.88, p < 0.001.

#### Time response effect on sex attribution

On average, participants in Experiment 3 took 6.18 s to assign sex to the stimulus (min = 1.51; max = 29.28). However, there were significant differences according to the type of displayed picture (i.e., male, female, neutral) (F = 12.71, p < 0.01). The shortest times (M = 5.92″) were observed with male stimuli (i.e., with male variables > 3; n = 47), whereas the longest times were observed when neutral stimuli (i.e., with a balanced co-presence of 3 male and 3 female variables) were displayed (M = 6.67″).

## Discussion

In the present study, we reported the findings of three experiments aimed at replicating Kessler and McKenna’s Overlay Study^15^. Unlike Kessler and McKenna, who administered stylized drawings of the human body (Supplementary Table S16), we administered the Sex/Gender Attribution Test for Adult^13^, created by digitally morphing the body of one human male and one human female model into realistic pictures (Supplementary Table S16). We assumed that, because evolved cognitive mechanisms are triggered on very specialized inputs^20^, more ecological and lifelike stimuli could have returned more reliable information about evolved cognitive processes. We expected, based on previous studies^13,15^, to find that primary sexual characteristics (genitals) would determine sex attribution (male/female) more than secondary/gender-linked sexual characteristics (short/long hair, male/female face, flat chest, breasts, narrow/wide hips, and body/no body hair), and that male sexual characteristics would determine sex attribution more than female sexual characteristics, with a significantly stronger effect of the penis compared to the vulva, *ceteris paribus*. The results have substantially reconfirmed the results obtained in the previous studies^13,15^. To facilitate the reading of the data, already discussed experiment by experiment above, we have reported the main results, in synoptic form, in Table 1.

**Table 1:** Synoptic table of the main results obtained in the three studies (Experiments 1, 2, and 3).

	Experiment 1	Experiment 2	Experiment 3
Participants (N = 1706)	n = 897	n = 30	n = 779
	Mean age = 21	Mean age = 21	Mean age = 22
	Females = 54.7%	Females = 70%	Females = 57.9%
Kinsey Scale	Heterosexual = 83.7%	Heterosexual = 50%	Heterosexual = 88.7%
Political opinions effect on sex attribution	Left-wing: lower percentage of female sex attribution	No difference	No difference
Cronbach’s alpha	α = 0.90 sex attribution	α = 0.70 sex attribution	α = 0.86 sex attribution
	α = 0.99 confidence	α = 0.99 confidence	
	α = 0.99 pleasantness		
Male attribution (vs female attribution)	60% all stimuli	58.1% all stimuli	58.8% all stimuli
	84.4% male stimuli	82.0% male stimuli	82.7% male stimuli
	35.3% female stimuli	34.3% female stimuli	35.1% female stimuli
	60.1% neutral stimuli	57.8% neutral stimuli	58.6% neutral stimuli
	83.3% neutral/male	81.4% neutral/male	80.1% neutral/male
	63.2% neutral/female	65.3% neutral/female	62.6% neutral/female
	87.7% penis exposed	88.4% penis exposed	84.3% penis exposed
	69.6% vulva exposed	73.3% vulva exposed	68.5% vulva exposed
Confidence (certain = score 7)	27.1% penis exposed	27.1% penis exposed	Not applicable
	20% vulva exposed	19.3% vulva exposed	
Confidence (uncertain = scores 1–6)	78.6% on female attribution	80.6% on female attribution	Not applicable
	73.2% on male attribution	74.5% on male attribution	
Unpleasantness (score 1)	Neutral stimuli = 38.6%	Not applicable	Not applicable
	Female stimuli = 31.3%		
	Male stimuli = 30.5%		

When the penis was apparent in a picture, the participants attributed male sex significantly more often (84.3–88.4 per cent) than female sex when the vulva was apparent (69.6–73.3 per cent). In other words, when male external genitalia were exposed (not covered by clothes), the odds of male sex attribution were 5.688 compared to 1.823 female attribution when female genitalia were exposed (Experiment 1). In addition, the certainty in sex attribution was reported to be greater when participants had attributed male versus female sex (Experiments 1 and 2). Furthermore, the participants attributed male sex to neutral stimuli 2.34, 2.83, and 2.17 times more often when the penis was displayed than when the vulva was shown in the Experiments 1, 2, and 3, respectively.

All findings had a strong statistical significance (Experiments 1, 2, and 3, p = < 0.001), substantially confirming those found by Federici and colleagues^13^ with the same realistic stimuli (SGAT-A) and by Kessler and McKenna^15^ with stylized drawings of the human body (Overlay Study).

Although all the sexual characteristics significantly contribute to the attribution of sex, female attribution appears to be triggered only when every other male cue has been excluded (Experiments 1 and 3). In other words, the presence of female sexual characteristics reduces the probability of assigning male sex. Therefore, all other things being constant, a female cue is recognized as such only in the absence of male cues. Whereas a male sex cue most likely equals male, a female cue equals female with much less probability, confirming Federici et al.’s^13^ and Kessler and McKenna’s^15^ findings.

The most salient variables were the penis followed by masculine face. The face assumed a prevalent role along with the flat chest and breast only when the external genitalia were covered by jeans (Experiments 1 and 3). The male face is an excellent predictor of male sex attribution^21,22^ and, if associated with the penis can overshadow all other female cues (face or vulva)^13,15^. For instance, in Experiment 1 the odds ratio (OR) of congruent sex assignment equals 3.209 in the presence of a masculine face and penis, with all the other characteristics being of a feminine type (Table S7, Model 00). Secondary sexual characteristics affect sex attribution only when primary ones are covered. In this case, gender-linked cues (long/short hair), and mainly the face also assumed a relevant role in orienting participants’ choices. These findings have confirmed what other similar studies found^13,15,23^: the penis and male sexual characteristics make the difference in sex recognition.

The salience of male versus female sexual characteristics suggests that the psychological mechanism does not operate on a dichotomous concept and binary sex categorization, but rather to solve an adaptive problem of avoiding at all costs a false negative by detecting a female when it is male^24^. In fact, for all individuals (e.g. infants, females), the risk of socializing with a male is greater than with a female, because male individuals tend to be physically stronger and much more aggressive. From this perspective, to mistake a female when it is a male is potentially more dangerous than the opposite for human survival. Therefore, a subtraction is applied to the higher danger condition (male) to get to the lack-of-danger condition (non-male). In other words, to survive, it is much more convenient to make a wrong female than a wrong male sex attribution. These errors of judgment are determined by cognitive mechanisms evolved by natural selection that “occurred despite the fact that subjects were encouraged to be accurate and were rewarded for the correct answers”^25^.

In Experiment 3, we have also examined the time taken by participants to attribute sex after each stimulus was displayed. As expected, the shortest times were observed with masculine stimuli (i.e., with male variables > 3; Supplementary Tables S16 and S17), whereas the longest times were observed with neutral stimuli (i.e., with a balanced co-presence of 3 male and 3 female variables; Supplementary Tables S16 and S17). Results showed that the more the stimuli were biased toward a binary sex (male/female vs neutral stimuli) the shorter the latency to sex attribution was computed. Then, stimuli with most male variables take less time and cognitive effort^16^ than any other. This result mirrors those by Simpkins^22^, who showed participants pictures of parts of real human faces, created by photographing 28 people who varied by sex, ethnicity, and age, on the grounds that the face is “usually the first source of information available about a person”^21^. The study concluded with a significant disproportion between the times in which the participants attributed male sex to the stimuli compared to times in which they attributed female sex to the stimuli. This was similarly demonstrated in the study by Wenzlaff and colleagues^23^, which replicated Kessler and McKenna’s study using eye tracking on digital reproductions of original stimuli. Participants gazed longer when they attributed female sex when a penis was present, than when they attributed male sex with a vulva shown. This is indicative of higher cognitive effort and more difficulty in ignoring the penis as opposed to the vulva. Attributing female sex when the individual might be a male requires a more careful and effortful attentional and decision-making process that also involves inhibiting the cognitive bias mechanism of male preference.

Experiment 2 also provided us with pilot data on cross-cultural differences on sex attribution. In order to test whether the results obtained in Experiment 1 were due to the Western cultural context on gender representation, in the second study Chinese students were recruited. All results substantially replicate those of Experiment 1. Although only first-time students moving to the West with very basic knowledge of English and almost no knowledge of Italian language were selected, nevertheless we cannot claim that they were representative of a Chinese cultural purity. In a globalized world, and particularly among the millennial generation, Western cultural influences extend to and inform Chinese young people in several ways (e.g. through the Internet) just as Chou^26^ argued in his book on homosexuality. Certainly, this phenomenon was known to us even before we conducted Experiment 2. But this pilot result prompts us to investigate further (e.g. with more extensive cross-cultural comparison) how far the absence of differences in sex attribution is due to a globalization of gender attitudes and stereotypes or to metacultural determinants. In other words, comparing different cultures allows us to investigate whether the process of sex attribution can be ascribed more to human universals^14,27^—that is, to the effect of evolved psychological mechanisms—rather than to the influence of memes. The fact that the young Chinese participants behaved similarly to those of the two Italian samples in attributing sex to stimuli from Western human models allows us to infer that the psychological mechanism of sex recognition may be metacultural and precede any form of ethnic differentiation. In the Pleistocene, our ancestors must have already been able to reliably proceed, being adaptively successful in recognizing the sex of conspecifics despite the individual phenotypic variation. We do not have a recognition mechanism that can differentiate a chicken from a hen, because it is not essential to our fitness. But we certainly needed to know how to discriminate the sex of a Neanderthal, Denisovans, or Homo sapiens before any ethnic (cultural) differentiation^28^. Another limitation of Experiment 2 is related to the fact that the results were based on a small-scale sample, not generalizable to be representative of another culture (Chinese); nevertheless they encourage us to continue our cross-cultural research.

Overall, the study supports the assumption that thinking a person is a male rather than a female is more likely and quicker, *ceteris paribus*, because maximizing male sex attribution reduces the risk of a false negative. We read these results not by limiting ourselves to an ethnomethodological perspective, as Kessler and McKenna did in the 1970s, but by integrating this with assumptions from evolutionary psychology and cognitive science, according to an Integrated Causal Model stating that “the distinction between the biologically determined and the nonbiologically determined can be seen to be a nondistinction”^29^.

The adaptive strategy, evolved in a psychological architecture of mind, neither excludes nor minimizes the cultural gender construction. Human minds and behavior, human artifacts and culture are.

all biological phenomena—aspects of the phenotypes of humans and their relationships with one another. The rich complexity of each individual is produced by a cognitive architecture, embodied in a physiological system, which interacts with the social and non-social world that surrounds it^29^.

This suggests that Kessler and McKenna’s^15^ argument from a constructionist point of view that “‘[sex] assignment’ and ‘gender construction’ may be synonymous”^15^ does not contradict the biological outlook according to psychological adaptive mechanisms evolved to respond to specific problems raised by the environment affecting human sex attribution. The fact that, in a phallocentric culture, a penis makes somebody more often a male person rather than a female one does not negate the fact that these cultural constructions were guided by an adapted mind^1^. There is no doubt that, in patriarchal cultures, the female role is derived from the space left free by the male role, though still under patriarchal control. So we can read biological cues as cultural: “the only sign of femaleness is an absence of male cues”^15^. However, this does not contradict that what culture has expressed, strengthened, sedimented, socially stratified, and handed down through cultural products and memes may have evolved from cognitive processes that have guaranteed human survival^30–34^. In case of ambiguity or complexity in the detection of sex cues, a cognitive bias has saved humans from a risky encounter with an aggressive male^24,35,36^. Cultural contents (e.g. phallocentrism, patriarchalism, androcentrism, etc.) cannot precede those psychological mechanisms that had produced them. This is not to say that culture only echoes mental contents. Alexander^37^ summarizes this point well as follows: “I have not suggested that culture precisely tracks the interests of the genes—obviously this is not true—but that, in historical terms, it does so much more closely than we might have imagined” (p. 142).

Once produced, the culture constitutes part of that environment within which the mechanisms of natural selection evolve. And since not only does the environment select the individual, but the individual modifies the environment, culture cannot completely introduce content that goes beyond the boundaries of those cognitive constraints within which all variations are possible and learnable. Otherwise, the content would be unlearnable, inexperienced and, therefore, without effect on the individual behavior and the phenotypic evolution.

How could we have evolved without developing a computational cognitive system specific enough to solve the problem of sex attribution in an infinite combinatorial variety of phenotypic and genetic variables? Just by combining six variables of primary and secondary male and female sexual characteristics and two articles of clothing, we produced 120 stimuli. Combinatorial explosion refers to the fact that with each new degree of freedom added, or dimension, or choice added, the total number of alternative possibilities quickly explodes. If only we were to combine the SGAT-A variables with other well-known variables that influence sex recognition—such as tone of voice, body posture and gait, social status, and so on^38^—the alternatives would soon multiply endlessly. Yet, each of us solves this “frame problem” without great difficulty or cognitive effort, in a matter of milliseconds^6^. This makes us lean toward a domain-specific mechanism that cooperates and competes with other evolved mechanisms of our adapted mind that have evolved for adaptive success and ensured good fitness in the Pleistocene.

## Method

In this section we provide details about the methodological aspects concerning the three experiments (i.e., participants, material, and procedure).

### Participants

#### Experiment 1

The SGAT-A was administered to 897 Italian Caucasian adults; only 1.1% (10 individuals) did not answer the test. Sex as assigned at birth was female for 598 (67.4%) respondents and male for the remaining 289 (32.6%) respondents. The median age was 21 years (min = 18; max = 90). The majority were undergraduates (52.2%; 54.7% females) (Supplementary Table S1). Of the participants, 95.3% (97.4% females) identified themselves in the man/woman binary gender identity among the 58 gender identity options (Supplementary Table S2). According to the Kinsey Scale, 83.7% (82.6% females) affirmed that they were exclusively heterosexual, 1.1% (0.7% females, 2.1% males) were exclusively homosexual, and 3.0% (3.0% females) were bisexual. Moreover, 8.9% (10.4% females, 4.8% males) affirmed to be predominantly heterosexual but with occasional homosexual components.

#### Experiment 2

The SGAT-A was administered to 30 Chinese university students (sex as assigned at birth: female = 70%). The median age was 21 years (min = 18; max = 30). Regarding the level of education, only one participant stated that they had not obtained a secondary school diploma, while 19 (63.3%) had reached a secondary school diploma (high school), and 10 (33.3%) had attended university up to a 3-year degree (Supplementary Table S8). Of the participants, 21 (69.9%) identified themselves in the man/woman binary gender identity among the 58 gender identity options, while the remaining 9 (about 30%) did not want to answer the question (Supplementary Table S9). According to the Kinsey Scale, 15 (50%) participants affirmed that they were exclusively heterosexual, 9 (30%) predominantly heterosexual but in some circumstances homosexual, one predominantly heterosexual but with a strong homosexual component, 4 (13.3%) essentially bisexual, and one exclusively homosexual.

#### Experiment 3

The SGAT-A was administered to 779 adults. Sex as assigned at birth was female for 451 (57.9%) respondents and male for the remaining 328 (42.1%) respondents. The median age was 22 years (min = 16; max = 89). The majority were undergraduates (47.8%; 45.9% females) (Supplementary Table S10). Of the participants, 97.2% (98.6% females) identified themselves in the man/woman binary gender identity among the 58 gender identity options (Supplementary Table S11). According to the Kinsey Scale, 88.7% (86.7% females) affirmed that they were exclusively heterosexual, 1.2% (0.7% females, 1.8% males) were exclusively homosexual, and 2.1% (2.9% females) were bisexual. Moreover, 5.5% (6.9% females, 3.7% males) affirmed to be predominantly heterosexual but with occasional homosexual components.

### Materials

A socio-demographic questionnaire was developed ad hoc to collect data on participants’ age, sex (as assigned at birth: male/female), gender identity (“I see/define myself a man”; “I see/define myself a woman”; plus Facebook’s 56 custom gender options for users who do not identify simply as “man” or “woman”), sexual orientation measured through the Kinsey Scale^17^, education, citizenship, religious beliefs, and political orientation.

Attribution of sex was performed through the Sex/Gender Attribution Test for Adult (SGAT-A). This test was designed by the authors (SF and AL)^13^; it includes 120 pictures of frontal human nudes created by combining parts of two original photographs of one male and one female model: six from the male (short hair, male face, flat chest, narrow hips, penis, and body hair) and six from the female (long hair, female face, breast, wide hips, vulva, and no body hair) model, plus two items of clothing (pants and t-shirt) (Supplementary Tables S16 and S17). The two original photographs of one male and one female model (Supplementary Table S16, red-framed stimuli 1_F1 and 64_M1) were the original pictures bought from the website http://www.3d.sk with a perpetual, non-exclusive, non-transferable worldwide license to use the content for the permitted uses. The other 118 stimuli have been created with the use of the software Adobe Photoshop 14. The use of Photoshop worksheets and tools such as “Magic Wand” and Lasso” as well as the copy and paste function made it possible to extract and combine the twelve human physical parts and two items of clothing according to a combinatorial calculation (Supplementary Table S17). An example of the use of Photoshop for stimuli design is provided in Supplementary Table S18.

Those stimuli with a majority of male variables (> 3) were coded with the letter “M” (47 male stimuli), those with a majority of female variables (> 3) with “F” (47 female stimuli), and those with a balanced co-presence of male (= 3) and female (= 3) variables were coded with “N” (26 neutral stimuli). M, F, and N refer only to the quantitative distribution of the variables in each stimulus (i.e., as the figures were depicted and manipulated) not to an evaluation of the biological sex of the figure represented in the stimulus. For each stimulus presented through an Internet platform, the participants were asked to assign the male or the female sex (“According to you, is the subject in the picture male or female?”). In addition, participants in Experiments 1 and 2 were asked to indicate on two different 7-point Likert-type scales the degree of confidence with regard to the sex attributed to the stimulus (“How confident do you feel about the answer you just gave?”) and participants in Experiment 1 were asked about the pleasantness of the picture (“How pleasant is the picture you have just seen?”). Both scales were anchored by 1 = not at all and 7 = totally; higher scores indicate greater levels of confidence/pleasantness.

For Experiment 2, both the socio-demographic questionnaire and the questions following the SGAT-A’s 120 pictures were translated to Chinese ideographic language. The translation was performed by a native Chinese speaker, magistral student to the University of Perugia. The chair of Sinology of the University of Perugia performed the back translation according to the guidelines of Beaton et al.^39^ Unlike Experiment 1, the question on the degree of pleasantness of each picture was eliminated, because of the difficulty in rendering the concept of pleasantness in Chinese in a manner comparable to that in Italian.

For Experiment 3, the response time from the presentation of the stimulus to the sex categorization (male/female) was computed and the questions about the degree of confidence and pleasantness were eliminated, to speed up administration time and the fluidity of the stimulus presentation.

#### Procedure

The study was approved by the Committee of Bioethics of the University of Perugia, protocol no. 2019-34. The observational study was carried out with full respect for the dignity of the human being and his/her fundamental rights, as dictated by the Declaration of Helsinki and the rules of Good Clinical Practice issued by the European Council. Informed consent was obtained from all individual participants included in the study. The task was administered individually to each participant in rooms located at the University of Perugia’s campus, set up with desks and chairs, with personal computers (PCs) dedicated exclusively to experimentation and protected by a password. After the participant signed the information sheet and the informed consent, the computer was switched on and the http://www.qualtrics.com platform was started. At this point, the investigator left the room and advised the participants that they could call them back at any time by ringing a reception bell placed on the desk. The Internet platform then provided the participants with the socio-demographic questionnaire and, afterward, the 120 stimuli of the SGAT-A in random order. The online administration, in total, took about 40 min to complete.

In Experiment 2, the mixed logit models were not estimated because of the reduced sample size. Participants were recruited among those attending the “Marco Polo” and “Turandot” programs of the University for Foreigners of Perugia. These programs are addressed to Chinese students who intend to achieve a university degree in Italian universities and Italian academic institutions of higher education in art and music. Only those students with a stay in Italy not exceeding 4 months and with a very basic knowledge of Italian and English were recruited. Information about the experiment was given to them in the Chinese language, either in writing or with an interpreter. In Experiment 3, the response times were calculated on the latency time taken by the respondents from the moment in which the stimulus appeared on the webpage until the moment in which they, after having assigned the sex (male/female), clicked on the “next” button at the bottom of the webpage. By clicking on the “next” button, a new stimulus appeared and a new calculation of the latency time started. The response times are measured in seconds.

## Supplementary Information

Supplementary Tables.

## Data Availability

Data for all experiments are posted at 10.13140/RG.2.2.20702.92482. The materials used in these studies are widely available.
